# Definition of icteric interference index for six biochemical analytes

**DOI:** 10.11613/BM.2023.020702

**Published:** 2023-06-15

**Authors:** Ruth Cano-Corres, Gemma Sole-Enrech, Maria Isabel Aparicio-Calvente

**Affiliations:** Clinical laboratory, Biochemistry Department, Parc Taulí Research and Innovation Institute Foundation (I3PT), Sabadell, Spain

**Keywords:** icterus interference, biochemical analytes, icterus

## Abstract

**Introduction:**

Icterus, if not detected, can affect the validity of results delivered by clinical laboratories, leading to erroneous results. This study aims to define bilirubin interference for some biochemical analytes and compare it with the manufacturer’s data.

**Material and methods:**

Serum pools prepared with outpatients’ samples were spiked with increasing bilirubin concentration (Merck, reference14370, Darmstadt, Germany) up to 513 µmol/L in order to evaluate the bias for the following biochemical analytes: creatinine (CREA), creatine kinase (CK), cholesterol (CHOL), gamma-glutamyltransferase (GGT), high-density lipoprotein cholesterol (HDL), and total protein (TP). For each analyte, six pools of different concentrations were prepared. Measurements were made employing Cobas 8000 analyser c702-502, Roche Diagnostics (Mannheim, Germany). This study employed a study procedure defined by the Spanish Society of Laboratory Medicine.

**Results:**

Obtained bilirubin concentrations producing a negative interference were 103 µmol/L for CHOL, 205 µmol/L for TP and 410 µmol/L for CK, but only for CK values less than 100 U/L. Bilirubin concentrations lower than 513 µmol/L do not produce interference for HDL and GGT. Finally, for the studied bilirubin concentrations, there is no interference for CREA higher than 80 µmol/L.

**Conclusion:**

Icterus interferences have been defined for each analyte, observing differences compared to data provided by the manufacturer. The evidence indicates that each laboratory should evaluate icteric interferences to ensure the high quality of the delivered results, thus benefiting patient care.

## Introduction

In clinical chemistry, interference is defined as a cause of clinically significant bias in the measured analyte concentration because of another component or property of the sample (*1*). Altered results could result in repeating tests, wrong diagnoses, and inappropriate patient treatment. On the other hand, the management of these results is time-consuming for the laboratory.

Several endogenous interferences have been described. Icterus is one of the most frequent endogenous interferences in clinical laboratories, caused by the presence of bilirubin (*2*, *3*). Mainali *et al.*, described in their study that 0.14% of clinical chemistry assays presented bilirubin interferences (*4*). The other most common endogenous interferences are haemolysis, which causes the release of cellular content, and lipaemia, because of the produced turbidity.

Bilirubin alters the measurement of analytes primarily through chemical and spectrophotometric interferences. Its chemical interference is manifested through its reaction with hydrogen peroxide, the intermediate component of some chemical reactions. Some analyte measurements interfered by bilirubin in this way are: cholesterol, uric acid, triglycerides and glucose. In cases of colorimetric enzymatic test for cholesterol and for glucose measured employing glucose oxidase, the middle step of the chemical reaction consists of the formation of hydrogen peroxide. In the last step, this hydrogen peroxide reacts with phenol and 4-aminoantipyrine producing a red colorant (*5*-*7*). In the case of uric acid and triglycerides, also the middle step of the reaction produces hydrogen peroxide, which will react with aminophenazone and other compounds producing a red colorant (*8*, *9*).

In all these cases, for icterus samples, the hydrogen peroxide will react with the bilirubin being consumed, and not disposable for the final chemical reaction.

On the other hand, for glucose measured with hexokinase method, as hydrogen peroxide doesn’t take part in this method, this assay is not altered by bilirubin, demonstrating that interferences are method dependent (*10*).

A spectrophotometric interference is manifested when the interfering substance has properties similar to the analyte, such as fluorescence, colour, light scattering, elution position, or electrode response detected and measured. Bilirubin presents high absorbance between 340-500 nm. That is why colorimetric assays of biochemical analytes taking absorbance measurements at these wavelengths could be affected, for example, the colorimetric test of alkaline phosphatase or Jaffe creatinine assay (*11*). In the last one, the formation of the coloured creatinine-picrate complex is measured by absorbance readings taken around 500 nm. Under the alkaline conditions of the reaction, bilirubin is oxidized to biliverdin, which causes negative interference by decreasing absorbance of the sample at 500 nm (*11*). Another example of this type of interference is the method for alkaline phosphatase measurement. It is based on the formation of the compound p-nitrophenol, which is measured by absorbance readings taken around 405-480 nm (*12*).

Analytical interferences are method dependent and not universal. For this reason, it is important that each laboratory knows the interferences affecting their biochemical analytes, and the mechanism of the interference.

In the past, the presence of icterus was assessed by visual inspection, a laborious and subjective method (*13*). Now, analysers incorporate systems for detecting interferences based on the spectral characteristics of haemoglobin, bilirubin and turbidity, and enable the automatic detection of interferences, increasing objectivity, sensitivity and speed. These analysers report three warning indexes (HIL: H - haemolysis, I - icterus, and L - lipaemia), which are calculated taking into account absorbance values obtained in the sample at a certain wavelength (*14*). The icteric and haemolytic index are equivalent to bilirubin and haemoglobin concentrations. Lipaemia can generally be measured without overlap from haemoglobin or bilirubin by taking a primary wavelength of 650 nm or higher, and it correlates with larger lipoprotein particles (*11*).

To establish the value of bilirubin concentration over which the studied interference is considered significant, the maximum permissible error (MPE %) must be defined.

Some authors have described differences between the icteric interference provided by the manufacturers and the experimental data obtained in the laboratory, especially for some biochemical analytes such as creatinine (CREA), creatine kinase (CK), gamma-glutamyltransferase (GGT), cholesterol (CHOL), high-density lipoprotein cholesterol (HDL) and total protein (TP) (*15*, *16*).

The study aims were: i) to establish the bilirubin concentration interfering with the previously defined biochemical analytes according to the study protocol; ii) to define the type of interference: the interference produces falsely elevated or decreased results; iii) to compare the obtained bilirubin concentration that causes interference with that given by the manufacturer.

## Materials and methods

### Materials

This study was performed between February and June 2022. Blood samples used in this study were obtained from outpatients referred to the laboratory for blood testing. Samples were drawn in gel separator tubes without anticoagulant (Vacuette Z serum separator clot activator, Greiner Bio-One, Kremsmünster, Austria). After laboratory testing, the remaining serum samples were used for study purposes. No additional blood sampling was needed for this research. Patients data were kept anonymous.

The biochemical analytes studied were CREA, CK, CHOL, GGT, HDL and TP.

This study has been designed employing a study procedure defined by the Spanish Society of Laboratory Medicine (SEQC-ML), which is, in turn, based on CLSI document EP7-A2 (*1*, *17*). The Hospital’s ethics committee approved the study as clinical research.

### Methods

Analyte measurements were performed using a Cobas 8000 c702-502 automated analyser of Roche Diagnostics (Mannheim, Germany). The specific methods used for each analyte are shown in Table 1.

**Table 1:** Measurement methods and pool concentrations of analytes used for determination of bilirubin interference

		**POOL CONCENTRATIONS**						**MPE (%)**	
**Biochemical analytes (units)**	**Measurement method**	**POOL** **1**	**POOL** **2**	**POOL** **3**	**POOL** **4**	**POOL** **5**	**POOL** **6**	**SEQC-ML 2022**	**Roche**
CK (U/L)	Colorimetric test Activator N-acetyl cysteine	20	50	100	200	500	1000	± 11.3%	10%
CHOL (mmol/L)	Colorimetric enzymatic test. Cholesterol oxidase, esterase, peroxidase	2.1	3.1	3.9	5.2	6.7	7.8	± 4.3%	10%
CREA (µmol/L)	Colorimetric compensated test based on the Jaffé method	44	80	111	150	177	221	± 7.4%	10%
GGT (U/L, 37ºC)	G-glutamyl-carboxy-nitroanilide - IFCC Ref. Proc., Calibrated	25	50	100	150	250	500	± 9.4%	10%
HDL (mmol/L)	Direct method, particles elimination and cholesterol esterase reaction	0.52	1.04	1.3	1.55	2.07	2.59	± 11.1%	10%
TP (g/L)	Colorimetric test, Biuret	40	50	60	75	85	100	± 3.5%	10%
CREA – creatinine. CK - creatine kinase. GGT - gamma-glutamyltransferase. CHOL – cholesterol. HDL – high density lipoprotein cholesterol. TP - total protein. SEQC-ML - Spanish Society of Laboratory Medicine. IFCC - International Federation of Clinical Chemistry and Laboratory Medicine. MPE - maximum permissible error. MPEs were obtained from SEQC-ML and manufacturer (Roche, Mannheim, Germany).									

For each analyte, six concentration pools were prepared employing patients serum samples. For each pool, we selected 15 patient serum samples with the desired analyte concentration, and mixed 1 mL of each sample to obtain the pool. The analyte concentration was measured in each pool to ensure that the obtained concentration was the one desired (Table 1). All concentrations were included in the measurement interval. Haemolysis and lipaemic index were measured; only if both of them were under 15, pools were employed for the study (H 15 is equivalent to 0.15 g/L haemoglobin, L index has no units because it correlates with the turbidity of the sample).

### Protocol

First, a bilirubin solution (1026 µmol/L) (SOLBIL) was prepared with 30 mg of lyophilized bilirubin (Merck, Darmstadt, Germany) and 5 mL 0.1 M NaOH. For each pool, two solutions were prepared: i) base solution without interferent (BSWO): 4.75 mL pool + 0.25 mL water; and ii) base solution with interferent (BSW): 4.75 mL pool + 0.25 mL SOLBIL. With these two solutions, the following eight dilutions were prepared: i) dilution 1 (bilirubin 0.0 µmol/L): 1.00 mL BSWO + 0.00 mL BSW; ii) dilution 2 (bilirubin 25.65 µmol/L): 0.95 mL BSWO + 0.05 mL BSW; iii) dilution 3 (bilirubin 51.3 µmol/L): 0.90 mL BSWO + 0.10 mL BSW; iv) dilution 4 (bilirubin 102.6 µmol/L): 0.80 mL BSWO + 0.20 mL BSW; v) dilution 5 (bilirubin 205.2 µmol/L): 0.60 mL BSWO + 0.40 mL BSW; vi) dilution 6 (bilirubin 307.8 µmol/L): 0.40 mL BSWO + 0.60 mL BSW; vii) dilution 7 (bilirubin 410.4 µmol/L): 0.20 mL BSWO + 0.80 mL BSW; and viii) dilution 8 (bilirubin 513 µmol/L): 0.00 mL BSWO + 1.00 mL BSW. The concentration of the studied biochemical analyte was measured in duplicate in each dilution and randomly, in the same analytical series. This means that the dilutions (16 aliquots) were processed randomly, to avoid carry-over. The bilirubin concentration was measured in each dilution in order to verify the declared concentration.

### Statistical analysis

**Figure 1:**
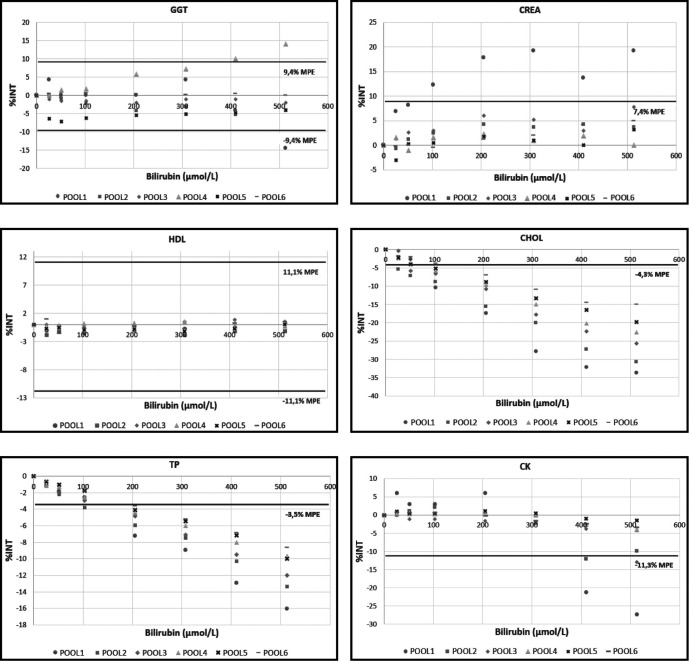
Interferograms representing Int% and %MPE (gray line) for each biochemical analyte. For GGT and HDL both the positive and the negative value of the %MPE are shown, and for the other analytes only one value of the %MPE is shown, depending on the sense of the interference. CREA – creatinine. CK - creatine kinase. GGT - gamma-glutamyltransferase. CHOL – cholesterol. HDL – high-density lipoprotein cholesterol. TP - total protein. MPE - maximum permissible error. %INT - Interference (%).

For each dilution, the average concentration of the two results (Cd) was calculated. The interference (Int(%)) was calculated as follows: Int(%) = 100 x (Cd - C1) / C1; where Cd denoted the average concentration in the studied dilution and C1 the dilution 1 concentration (no interferent). For each pool, an interferogram was represented, with Int(%) on the x-axis and the bilirubin concentration on the y-axis (Figure 1).

To establish the value of bilirubin concentration over which the studied interference is considered significant, we selected MPE (%) (positive or negative value) in the quality specification working paper of the Spanish Society of Laboratory Medicine 2022 (SEQC-ML) (Table 1) (*18*). Those MPEs differ from the values employed by the manufacturer, which are about 10% for all analytes. Bilirubin concentration in the first dilution presenting Int(%) higher than MPE (%) was considered an interferent. For data evaluation, Microsoft Excel 2016 (Microsoft, Redmond, USA) was employed.

## Results

Bilirubin concentrations lower than 513 µmol/L did not interfere with HDL and GGT measurements. Bilirubin concentrations higher than 103 µmol/L for CHOL and 205 µmol/L for TP interfered with their measurements, resulting in falsely decreased results. Bilirubin concentrations higher than 410 µmol/L interfered in a negative way in the measurement of CK, but only for CK values less than 100 U/L. Finally, there was no interference for CREA higher than 80 µmol/L.

Figure 1 shows the Int% (positive or negative) obtained for each pool’s dilution and biochemical analyte and the SEQC-ML MPE%. Table 2 shows the bilirubin concentration considered as interferent provided by the manufacturer, and the value calculated in our study.

**Table 2:** Obtained bilirubin concentrations considered as interferent in our study and for manufacturer

**Biochemical analytes (unit)**	**Bilirubin concentration (µmol/L)**	
	**Our study**	**Roche**
CK (U/L)	410*	1026
CHOL (mmol/L)	103	240
CREA (µmol/L)	> 513^†^	171
GGT (U/L, 37ºC)	> 513	342
HDL (mmol/L)	> 513	1026
TP (g/L)	205	342
*Only interfering CK lower than 100 U/L. ^†^only for CREA higher than 80 µmol/L. CREA – creatinine. CK - creatine kinase. GGT - gamma-glutamyltransferase. CHOL – cholesterol. HDL – high-density lipoprotein cholesterol. TP - total protein.		

## Discussion

Analytical interferences caused by preanalytical factors, such as icterus, are a significant source of error in clinical laboratory measurements (*19*).

Data obtained in our study are only in accordance with bilirubin interferences reported by the manufacturer for HDL. For CHOL and TP, lower bilirubin concentrations than the ones proposed by the manufacturer were considered interferent. For both cases, a clear negative interference has been observed. The study made by the manufacturer reported icteric interference only for CHOL 5.2 µmol/L while we studied concentrations from 2.2 to 7.8 µmol/L and, as it is shown in the interferogram, the bias is higher for lower CHOL concentrations. This fact, and that the %MPE selected is lower than the manufacturer one, could explain the differences. The same occurs for TP.

A negative interference was also detected regarding CK, but only for CK values lower than 100 U/L. Manufacturer made the study only for higher CK concentrations, where we did not find interferences. For GGT, higher bilirubin concentrations than the ones proposed by the manufacturer were considered interferent, although %MPE from SECQ-ML and manufacturer are similar.

Finally, contrary to what the manufacturer indicates, there is no bilirubin interference for concentrations of CREA higher than 80 µmol/L. In this case, a positive interference is detected. This work observed the bilirubin interference for six different CREA concentrations, while the manufacturer only tested the interference for CREA concentration of 80 µmol/L, just the value for which authors detected interferences. The manufacturer Jaffé chemical test is based on target determination to minimize interference by bilirubin. This mechanism intended to avoid the interference may be more effective for higher CREA concentrations.

We believe that our study, employing different analyte pool concentrations, provides important information because the study is conducted in a more comprehensive manner.

Alvarez *et al.* also studied the icteric interference in Roche Jaffé method, demonstrating that the method exhibits acceptable performance in the presence of icterus at icterus indexes above the manufacturer’s recommendations (*20*). Our results support this assertion; icterus seems to affect positively, but only the measurement of CREA for concentrations under 80 µmol/L. Roseri *et al.* also studied the influence of bilirubin for CREA measurement employing different methods, showing a bias of -1.4% for Jaffé method and emphasizing the poor specificity of this method (*21*).

Some other authors, like Ji and Meng, have studied the icteric interference for the same manufacturer (*15*). Contrary to our study, these authors employed the 10% MPE as manufacturers do. We believe that our criterion is more adequate because it depends on the biochemical analyte characteristics. For CHOL, they did not detect significant interference produced by bilirubin, but in our case, we detected that CHOL concentrations significantly decrease when bilirubin is present. These differences are observed for all CHOL concentrations considering our MPE (4.5%) and MPE (10%). On the other hand, Ji and Meng detected significant negative interference for HDL, but in our study, no interference was detected. As regards CK and TP, our data are similar.

Nicolay *et al.* also studied the relationship between the icteric index and the biochemical analytes variations (*16*). They report that CHOL and TP decreased significantly when bilirubin increased, as we observed in our study. On the other hand, they show significantly decreased CREA concentrations when our study shows a positive interference. They studied the enzymatic creatinine assay, which is likely the explanation for differing behaviour. This study shows no icteric interference for GGT, supporting our results.

Castaño *et al.*’s study, carried out many years ago but with the same methods as our study, also presented results in accordance with ours. For CHOL and TP they showed a strong negative influence of bilirubin and no influence for CREA (*22*).

Ali *et al.* studied lipaemic and icteric interferences for some biochemical analytes, including the ones in our study. This study employed Cobas 6000 automated analyser (Roche Diagnostics, Mannheim, Germany), which uses the same methods as Cobas 8000. They also found no bilirubin interference for GGT and a negative interference for CHOL and TP. No interference for high values of CREA was observed (*23*).

One of the strengths of our study is that we have employed the MPE defined by SECQ-ML 2022 (*18*). There are three models defined by the European Federation of Clinical Chemistry and Laboratory Medicine (EFLM) in the Milan Conference 2014 to state the MPE, depending on the analyte characteristics: based on clinical outcomes, on biological variations or in state-of-the-art (*24*). The SECQ-ML has defined the preferred model for each analyte and the recommended MPE in its documents. This criterion is stricter and more adequate than the one used by the manufacturer, which is about 10%. This is the distinguishing feature of our work. Moreover, another strength of our study is that lipaemia and haemolysis are measured in all pools, thus discharging these interferences. Our work studies the icteric interferences for biochemical analytes taking into account different analyte concentrations, while manufacturer and most of the previously described works only make the study for one concentration. For this reason, our work shows a more complete information, and this could explain the discrepancies between published data.

This study also presents some limitations. Only some biochemical analytes have been evaluated; in future studies, some other analytes could be included. The limit on the maximum bilirubin interference concentration tested was 513 µmol/L whereas the manufacturer used 1026 µmol/L, it is possible that some analytes can be measured without interference in samples with higher bilirubin concentrations than 513 µmol/L, however this was not evaluated in our study. This limit is chosen because it is the one determined by the SEQC-ML protocol, but it is not common to find higher bilirubin concentration in patient’s samples. In addition, the study design includes pipetting different volumes, which introduces error to the measurements.

The evidence provided indicates that each laboratory should evaluate icteric interferences to ensure the high quality of the delivered results, thus improving patient care. Each laboratory should define how restrictive they want to be when choosing their own MPE (%), and define their own interference index. In our laboratory, our own calculated icteric indexes will be implemented.

In conclusion, this study defines the bilirubin concentration interfering the measurement of six biochemical analytes (CK, COL, HDL, TP, CREA and GGT), and also describes the positive or negative trend of the interference. Finally, data obtained in our study are not fully in accord with data provided by the manufacturer. Our study confirmed manufacturer claims of icteric interference for HDL, manufacturer overestimates claims for CK, COL and TP, and underestimates claims for CREA and GGT.
